# Involving expert patients in antiretroviral treatment provision in a tertiary referral hospital HIV clinic in Malawi

**DOI:** 10.1186/1472-6963-12-140

**Published:** 2012-06-08

**Authors:** Lyson Tenthani, Fabian Cataldo, Adrienne K Chan, Richard Bedell, Alexandra LC Martiniuk, Monique van Lettow

**Affiliations:** 1 Dignitas International, Zomba, Malawi; 2 Department of HIV and AIDS, Ministry of Health, Lilongwe, Malawi; 3 Dalla Lana School of Public Health, University of Toronto, Toronto, Canada; 4 Department of Medicine, St. Michael’s Hospital and Institute of Health Policy, Management and Evaluation, University of Toronto, Toronto, Canada; 5 Sunnybrook Health Sciences Center, Toronto, Canada; 6 The George Institute for Global Health at the University of Sydney, Sydney, Australia; 7 Faculty of Medical Sciences, University College London, London, UK

**Keywords:** Task Shifting, Expert patients, Antiretroviral treatment, Malawi

## Abstract

**Background:**

Current antiretroviral treatment (ART) models in Africa are labour intensive and require a high number of skilled staff. In the context of constraints in human resources for health, task shifting is considered a feasible alternative for ART service delivery. In 2006, Dignitas International in partnership with the Malawi Ministry of Health trained a cadre of expert patients at the HIV Clinic at a tertiary referral hospital in Zomba, Malawi. Expert patients were trained to assist with clinic tasks including measurement of vital signs, anthropometry and counseling.

**Methods:**

A descriptive observational study using mixed methods was conducted two years after the start of program implementation. Semi-structured interviews were conducted with 20 patients, seven expert patients and six formal health care providers to explore perceptions towards the expert patients’ contributions in the clinic. Structured exit interviews with 81 patients, assessed whether essential ART information was conveyed during counseling sessions. Vital signs and anthropometry measurements performed by expert patients were repeated by a nurse to assess accuracy of measurements. Direct observations quantified the time spent with each patient.

**Results:**

There were minor differences in measurement of patients’ weight, height and temperature between the expert patients and the nurse. The majority of patients exiting a counseling session reported, without prompting, at least three side effects of ART, correct actions to be taken on observing a side-effect, and correct consequences of non-adherence to ART. Expert patients carried out 368 hours of nurse tasks each month, saving two and a half full-time nurse equivalents per month. Formal health care workers and patients accept and value expert patients’ involvement in ART provision and care. Expert patients felt valued by patients for being a ‘role model’, or a ‘model of hope’, promoting positive living and adherence to ART.

**Conclusions:**

Expert patients add value to the ART services at a tertiary referral HIV clinic in Malawi. Expert patients carry out shifted tasks acceptably, saving formal health staff time, and also act as ‘living testimonies’ of the benefits of ART and can be a means of achieving greater involvement of People Living with HIV in HIV treatment programs.

## Background

Lay health workers have the potential to be involved in the provision of HIV care through ‘task shifting’. With the severe constraints in human resources for health (HRH), there is an unmet need for HIV care in low and middle income countries, particularly in sub-Saharan Africa [1-3] . Increased health worker morbidity and mortality as well as increased demand for health care in this setting, has further strained the already compromised health system [1-4]. Like other health systems in sub-Saharan Africa where high HIV prevalence intersects with HRH constraints, Malawi (adult HIV prevalence rate of 14.5% [5]; 1 doctor: 26 nurses per 100,000 population [6,7]) employs a model of rapid antiretroviral treatment (ART) scale-up that can be labour intensive, requiring a high number of skilled staff. Task shifting has been increasingly employed in Malawi [1-4,8] to mitigate the HRH constraints, whereby tasks usually performed by doctors have been shifted to clinical officers and nurses, and lay health care workers known as health surveillance assistants in turn have taken on many of the tasks usually done by nurses [1].

Task shifting may also be envisaged as a means of achieving Greater Involvement of People with HIV/AIDS (GIPA) as advocated by the WHO and scientists [9-12]. Although the involvement of individuals living with HIV was first articulated over a decade ago by AIDS activist groups and by UNAIDS in 1999, little research has been done to assess the extent of the adoption of this principle. More recently, patient-centred initiatives started to involve HIV patients in routine care services in order to carry out specific tasks in the HIV care and treatment continuum, whilst providing additional health awareness advice and assistance to facility and home-based HIV services. To date, few studies have focused on exploring the feasibility and acceptability of such interventions. Some also questioned the ‘universal’ applicability of the ‘expert patient’ model, and emphasised the need to carefully consider how roles and relationships may shift as patients becomes assimilated into mainstream care systems [11]. The advent of ‘expert patient’ interventions in countries such as Malawi offers a lens into the operationalisability of such approach.

To increase the involvement of people living with HIV (PLHIV) and to respond to the current HRH crisis in Malawi, an expert patient program was launched in Zomba Central Hospital (South-East region) in 2006. An operational study was conducted to assess the performance and acceptability of expert patients in HIV care provision in Zomba Central Hospital.

### Study setting and description of intervention

Zomba district (population 670,500) is situated in the southern region of Malawi, and has one of the highest estimated adult HIV prevalence rates in the country (14.5%) [5]. The district is served by one Central Hospital and more than 20 rural health centers run by the Ministry of Health (MoH), and the Christian Health Association of Malawi. The district ART program, run by the MoH with the support of Dignitas International, a Canadian NGO, started in 2004 [13]. Through this programme, as of September2011, over 17,500 patients have been enrolled on ART.

In 2006, clinic staff were asked to identify areas of patient care where they felt a task shifting initiative might be helpful due to the rapidly increasing workload. The tasks identified included filing of patient level ART data, vital sign measurement and individual counseling of patients on how to take ART using a counseling checklist. Consequently, Dignitas International trained a small group of expert patients at the tertiary referral *Tisungane* HIV clinic at Zomba Central Hospital. Initially, 13 patients who were stable on ART and had been followed at *Tisungane* clinic for over six months were selected and agreed to join an expert patient training program based on their willingness and interest to support other people living with HIV. All of them had completed primary school education and some had completed their Malawi Junior CertificateExamination (a national standardized test conducted after two years of secondary education).

Expert patients were trained using the standardized two day Malawi MoH training course for ART counsellors (on ART initiation and ART adherence) as well as ART clerks (on use of standardized MoH registers and mastercards). In addition, through a one day didactic session and clinical mentorship, senior nurses trained and supervised the expert patients to assist with patient transfer, transport and light clinical tasks, specifically anthropometry and recording of vital signs *i.e.* height, weight and temperature. Additional briefing was also done on the ethics of interactions with fellow patients focusing on patient confidentiality.

Of the initial 13 patients, six left due to personal reasons and concerns regarding the sustainability of financial remuneration. Seven expert patients have worked in the *Tisungane* clinic since the start of the initiative.

Prior to 2008, the expert patients received non-financial compensation (rain gear, bicycles, lunchtime meal, Malawi MoH Home Based Care curriculum training and associated allowances). From 2008, expert patients were provided with a small stipend from *Paediatric AIDS Treatment for Africa*.

Expert patients were primarily supervised by the MoH senior nurse in charge (for vital signs), a nurse who specializes in adherence and psychosocial support (for group and individual ART counselling) and the MoH ART Data Clerk (for data management). Expert patients were included as part of bi-weekly clinic meetings for quality improvement and also attend their own bi-weekly meeting with a nurse supervisor. Over time, the level as well as nature of the tasks performed by expert patients has expanded. Some of their daily tasks include welcoming patients, organizing patient flow, taking vitals prior to entering the nursing rooms, and perform a rudimentary triage to send patients that appear to need more immediate attention directly to the clinical officer’s rooms.

The*Tisungane* clinic’sformal health care workers and auxiliary/lay health workers responsibilities at the time of study are listed in Table 1. The patient clinic volume at the time of study averaged 5,000 patient visits and 300 new patient initiations on ART per month.

**Table 1:** Role of health staff in Tisungane Clinic at time of study

**Health Cadre**	**Job Tasks and Responsibilities**
***Formal health care workers***	
Physicians	*1 Clinic Coordinator (Dignitas International) conducting specialist consultations on patients with complicated ART related issues or complex diagnostic cases; daily to bi-weekly clinical mentorship of clinical officers and nurses*
	*1 Pediatrician (Baylor International Pediatric AIDS Initiative) conducting specialist consultations on pediatric patients 2 days a week*
Clinicians	*3 Clinical Officers (Dignitas International) conducting patient consultations: opportunistic Infections (OIs), staging, ART initiation, primary HIV care, inpatient consults*
Nurses	*5 Nurses (3 Ministry of Health, 2 Dignitas International) supporting clinicians: prepare and counsel people for ART initiation, monitor ART recipients, manage drug supply and dispense medication, adherence counseling, manage some minor ART toxicities, administer vincristine for Kaposi’s Sarcoma patients; supervise data collection and defaulter tracing*
***Auxiliary health workers and Lay Health care providers***	
Patient Care Attendants	*1 ART Clerk, 1 ART Clerk/Counselor, 3 Nutrition Counselors and Adherence Counselors (Ministry of Health): collect and organize MOH mastercards and register patients on ART, nutrition assessments and counseling and administration of therapeutic feeding, counseling for ART initiation, adherence counseling*
Health Surveillance Assistants	*District Environmental Health Office (Ministry of Health) coordinates Health Surveillance Assistants to assist with defaulter tracing in Zomba District*
Expert Patients	*7 Expert Patients (Dignitas International): patient escorting and transport, assist ART Clerk, ART initiation and adherence counseling, light nursing tasks (vital signs, anthropometry – heights, weights, MUACs), triage and patient flow, translation*

## Methodology

### Study design

A descriptive observational study using qualitative and quantitative data collection tools was conducted in November and December 2008, two years after implementation of the program at the ART clinic in Zomba Central Hospital.

All expert patients (seven) working at the ART clinic (Zomba Central Hospital) were included in interviews and observations. All formal health staff working at the same ART clinic (one physician, three clinical officers and two nurses) were invited to participate in semi-structured interviews. In addition, 20 ART patients were randomly selected to take part in semi-structured interviews. Every third patient in the queue to the nurse drug dispensing room was asked to participate in the study. Last, for a period of two weeks, all patients who received a counselling session conducted by an expert patient were invited to respond to a structured exit questionnaire. This questionnaire was conducted with a total of 81 patients.

### Data collection

The methodology included semi-structured interviews with expert patients, patients and medical staff; structured exit interviews with patients; structured observation during expert patients’ interactions with patients; and reassessment of anthropometry and vital signs information by a nurse.

The semi-structured interviews were conducted using an interview guide with seven expert patients, one medical doctor, three clinical officers, two nurses and 20 randomly selected ART patients to explore their perception of expert patients’ care provision. Open questions sought to elicit formal health care workers’ and general patients’ views of expert patient competency, contributions to and quality of service provision, as well as client-patient relationship.

The 81 structured exit interviews were conducted with patients directly after ART counselling sessions with an expert patient. Using a structured questionnaire, patients were asked what they had learned during the session and information they received about drug taking instructions, side effects and adherence. The questionnaire aimed to assess the type of information conveyed by expert patients, and if essential ART information was provided.

Structured observation during patient and expert patient interactions were conducted in order to establish how much time was spent in counselling as well as during the collection of anthropometric and vital signs information. The researcher measured the time of counselling sessions, and the time taken between the start of height, weight and temperature measurements and the recording of this information per patient.

Last, after patients were seen by an expert patient, a nurse was tasked to re-assess the measurements performed by expert patients (weight, height and temperature) on 93 patients.

### Data management and analysis

Each study participant was assigned an anonymised unique identifier. Each interview was transcribed in full and translated (from Chichewa to English). Qualitative data were managed and analysed using NVIVO software. A coding system was established based on analytical categories, and themes were identified in relation to perceptions about the contribution of expert patients to the HIV program. Coding categories included perceived expert patient competency, service provision, provider/client relationship, quality of care and contribution to HIV service delivery.

Quantitative data were analyzed using SPSS software. Summary statistics were derived for continuous variables while categorical variables were presented as proportions. Mean differences in readings of weight, height and temperature between the expert patient and the nurse’s readings were provided, along with their standard deviations and 95% confidence intervals (95% CI).

All study participants gave informed consent to participate in study. This study received approval for publication of results from the National Health Science Research Committee in Malawi.

## Results

### Profile of expert patients and patients interviewed

The average age of the seven expert patients (five females and two males) was 35 years – ranging from 27 to 45 years. The length of time they were on ART ranged from three to six years. They all have children, all of whom are HIV uninfected, except for one whose mother was started on ART after the child’s birth. All expert patients lived within walking distance from the hospital. Table 2 outlines demographic information about each expert patient.

**Table 2:** Expert patient demographics at time of study

	**Age**	**Sex**	**Duration on ART**	**Marital Status**	**Spouse serostatus**	**Number of Children; serostatus**	**Education Level**	**Primary tasks as expert patient**
EP1	27	F	3 years 4 months	Spouse	HIV-infected	3; HIV uninfected	Primary school	Vitals and Triage, Counseling
EP2	34	F	3 years 1 months	Widow	HIV-infected	1; HIV uninfected	Junior Certificate	Vitals and Triage, Counseling
EP3	39	F	3 years 2 months	Spouse	HIV-infected	2; HIV uninfected	Primary school	Vitals and Triage, Counseling
EP4	34	F	3 years 7 months	Widow	HIV-infected	3; (1 HIV infected; 2 HIV uninfected)	Junior Certificate	ART Clerk Assistant
EP5	35	F	4 years 4 months	Widow	HIV-infected	1; HIV uninfected	Junior Certificate	Vitals and Triage, Counseling
EP6	34	M	2 years 10 months	Spouse	HIV-infected	2; HIV uninfected	Junior Certificate	ART Clerk Assistant
EP7	45	M	6 years 3 months	Spouse	HIV-infected	2; HIV uninfected	Junior Certificate	Counseling, Translation, ART Clerk Assistant

Almost two thirds (n = 66) of all patients interviewed were female and their average age was 34 years. Over two thirds (n = 69) of patients had received some primary education, a minority (n = 17) reported they had no education at all and few (n = 15) had at least two years of secondary education.

### Expert patient performance

The study documents that expert patients contribute to a number of essential tasks within the facility. They facilitate patient flow management and triage, take patients’ vital signs, temperature and heights/weights. In addition, expert patients conduct individual and adherence counseling for ART, and assist with record keeping.

In order to assess the competency of the expert patients in taking temperatures and anthropometric measurements, reassessment of 93 patients was carried out by a nurse. There were minor differences in all readings between the expert patients and the nurse. The mean weight difference between the expert patient and the nurse was −0.003 kg, SD 0.2 (95% CI −0.04-0.03); the mean height difference was 0.006 cm, SD 0.5 (95% CI −0.1- 0.1); and the mean difference in temperature readings was 0.06 °C, SD 0.6 (95% CI −0.06-0.2).

To assess whether essential ART information was conveyed during counseling sessions performed by expert patients, patients were asked on exit interview what the content of the session was. Based on un-prompted responses by patients counseled, the most frequently reported content of counseling sessions focused on ART provision, side effects, drug taking instructions, the need for treatment adherence, and the statement that ‘ARVs are not a cure for AIDS’.

When more specific questions were asked, almost all patients (79/81) stated that ART is taken for the rest of their life. All patients correctly stated that ART is taken twice a day. Sixty-one of the participants affirmed that only one pill is taken, and 18 stated that two pills are taken each time; the remaining two patients did not know how many pills they would need to take each day. Those who stated the need to take two pills also mentioned the need to take cotrimoxazole preventive therapy with the ART. All participants stated that sharing medication with others is not allowed. Eighty of 81 participants correctly mentioned that non-adherence to ART could have consequences to their health. Over half (44/81) of the participants mentioned drug resistance and 46% (37/81) mentioned that low immunity could lead to the occurrence of other (opportunistic) infections.

In addition, after counseling with an expert patient, most participants correctly stated at least three serious side effects that a person may experience while on ART as stated in the MoH standardized counseling guidelines. About 60% (49/81) mentioned vomiting, whilst few respondents mentioned diarrhea and abdominal pain. Upon inquiry about jaundice, 91% (74/81) stated that they had to see a clinician if experiencing jaundice; 9% (7/81) stated the need to continue taking the medication. All 81 participants stated that one needs to see a clinician if experiencing severerash and sores.

Based on program reports, expert patients carry out an average of 175 counseling sessions and 4865 patient vital signsrecording per month. Direct observations showed that a counseling session takes 15 minutes on average, whilst vitals and anthropometric assessment on each patient takes four minutes on average. The expert patients therefore carry out 368 hours of nurse tasks each month, which in absolute terms would translate into two and a half full-time nurse equivalents per month.

### Expert patient acceptability

All seven expert patients stated that they felt generally accepted by patients. However, expert patients felt they are viewed with some suspicion at first when they disclose to other patients that they are also on ART themselves. This trend seems to change with time and further interactions between patients and expert patients. One of the expert patients stated: *in the first days the patients could not believe that we are also HIV positive because they expected that people who are HIV positive are supposed to always look sick and have poor health* (Female expert patient, 34 years). Another expressed that *when they* [PLHIV] *believe that you are like them, they become close to you in order to receive comfort from you, and you end up understanding each other easily* (Male expert patient, 45 years).

Some expert patients expressed that they felt valued by patients for being a ‘role model’, or a ‘model of hope’, promoting positive living and adherence to ART. One patient mentioned: *I see how healthy she* [the expert patient] *is* (Female patient, 36 years). One of the expert patient added: *I tell them* [patients] *that all things are possible but it is good that they* [patients] *should follow the advice from the doctors and nurses. I tell them that if they do this, they can have a long life, like me* (Male expert patient, 34 years).

Overall, expert patients appear to be relatively well accepted as service providers by patients, mainly because expert patients were seen as more responsive to patients’ requests. A patient explained: *there is a big difference* [between expert patients and formal health care workers];*when you ask expert patients they answer you in a good manner and if they are not too busy they can even escort you to the place you want to go without any complaint (Female patient, 42 years).*

Observations and interviews revealed that patients were not always aware that the clinic staff member who they were seen by during consultations was an expert patient. Expert Patients appeared to disclose their status less frequently than expected to new patients compared to patients followed longer in the clinic. Patients were told by the expert patient themselves that they were also taking ART.

Confidentiality was particularly valued by patients, who saw expert patients as reliable sources of support. One of the patients expressed that because she knows that the expert patient can keep her *secret because they know the problem we are facing when you are taking these ARVs (Female patient, 34 years).*

Patients also expressed feeling less discriminated against, and treated more fairly by expert patients. *When they* [expert patients] *have seen their relative or they have a friend who also wants to receive ARVs,* said one of the patients, *they don’t put them in front; they do according to their number in the queue* (Male patient, 33 years).

Patients expressed that they feel closely related to the life experience of expert patients. *Expert patients are people who are also taking these ARVs,* said one of the patients, *so they differ from other health care workers just because expert patient take ARVs they are able to understand you* [better] *than others* (Female patient, 15 years).

Some health care workers expressed that expert patients were efficient as they could delegate a number of tasks to them. *Their* [expert patients’] *work is good because it’s time saving and I don’t see any problems in how they care for the patients” (Female Nurse, 51 years).* However, other health care workers expressed that the performance of expert patients is not optimal, particularly in recording vital signs. One of the clinical officers stated that he accepts *a lower standard* [of care] *to improve efficiency in terms of time* (Male Clinical Officer, 34 years).

## Discussion

This study examined the contribution of expert patients to an HIV program in an ART clinic in Malawi, to understand the performance and level of acceptability of patient involvement in formal HIV service delivery. Expert patients are living with HIV themselves and, at the time of study, were already on ART for several years. All expert patients had a partner living with HIV, but some were now widowed. They all live in low income areas around the hospital and have a similar level of formal education as the patients they support. The background and proximity of expert patients and patients’ illness and treatment experiences suggest they may be able to relate with each other on a more personal level.

This study shows that numerous tasks previously performed by nurses and patient care attendants were successfully taken on by expert patients involved in ART services. To some extent, expert patients represent a unique cadre of health workers in that they utilize both newly acquired task-shifted ‘health care’ skills, as well as personal experiences of illness and treatment.

Two recent studies from Kenya [14,15] examined the performance of expert patients who provided ART delivery and follow-up in remote communities with the use of an electronic decision support tool. These studies found that expert patients performed similarly to formal health professionals and that they could take on a wide range of tasks and perform them well, with positive outcomes for ART delivery and follow up of patients [14,15]. Another study from Mozambique shows that expert patients supporting treatment as part of a ‘community ART group’, may also help to improve retention in care and adherence to ART [16]. The Mozambique program also demonstrated that providing group counselling increased the efficiency and potential impact of expert patients. Our study showed that, similar to the previously published studies mentioned above, when assessing the performance of the expert patients compared to the nurses, there were minor differences in measurement of patients’ weight, height and temperature. These differences can be expected with two measurements made by different individuals regardless of their health-related training. In terms of counseling skills, we observed that most patients received essential information and adequate support, as judged by patients themselves.

Furthermore, expert patients were widely accepted by patients who valued the care they were given, and who portrayed expert patients as ‘role models’. This observation also suggests that, in their role as patient themselves, expert patients may also assist in improving relations between health providers and patients [17,18]. The positive perception of the role of the expert patient by patients themselves is further reinforced by the view that they are ‘like them’, as patients put it, and more likely to understand patients circumstances and respect confidentiality compared to other health workers. Similar to our findings, other studies have shown that people living with HIV like to receive information about HIV from other HIV positive persons [19].

In relation to other health staff, previous studies describe that formal health workers may initially be skeptical about expert patients, but that their reservations tend to diminish over time [12]. We show that formal health staff perceived the expert patients’ clinical competency to be somewhat inferior to the standard of care they provide. However, expert patients were explicitly accepted by nurses and clinical offers in the context of recurrent staff shortages in the HIV clinic.

Last, we argue that expert patients themselves benefit from working within the health care team, taking on a professional role whilst supporting others, and increasing their skills and knowledge on HIV care. Previous studies have also highlighted that the involvement of people living with HIV in HIV care is associated with increased quality of life and self-esteem [20,21], pride in being a part of the health care team [10] and decision making, disclosure of one’s own status and formation of more support groups [10]. However, concerns regarding the sustainability of expert patient-led interventions need further attention, as a number of expert patients in this setting left because they were unsure about future resources (such as volunteers’ stipend) being made available to them and to the expert patient program.

Some of the limitations of this study include the relatively small number of expert patients studied, and the short period for data collection, which may have precluded our ability to observe rare but important safety concerns, or potential institutional resistance. In addition, the awareness that they were being assessed might have affected the performance of expert patients. Because this study was a site-specific observational study in a clinic that benefits from additional NGO resources, results may not be generalizable to other settings. However, the results from this study mirror the results observed in similar studies conducted in other countries [14-16] suggesting overall positive performance and acceptability of expert patients working within ART services in sub-Saharan Africa.

## Conclusions

Task shifting, or delegating tasks to staff with lower-level qualifications, is currently considered a gateway to address HRH shortages and provide a means of expanding the roll-out of HIV treatment and care in resource-poor or HRH-limited settings [17,18,22,23]. Our study illustrates that expert patients successfully supported formal health care workers and helped reduce their daily workload. Expert patients in this study add value to the ART services; they not only carry out the shifted tasks acceptably thus saving formal health staff time, they also act as ‘living testimonies’ of the health related benefits of ART, and they contribute to increasing the involvement of people living with HIV in treatment and care programs.

Patient-centred interventions such as the expert patient program described in this study have only recently been pioneered in Malawi. Governments and NGOs are increasingly promoting expert patients as a useful and under-utilized human resource [24] particularly in low resource settings; this study adds to the growing body of evidence regarding the value of expert patients in a task-shifting environment, both in improving the quality and efficiency of HIV service scale-up.

Further investigation into the expansion and scope for new roles and tasks performed by expert patients should include a close examination of their potential to contribute to large scale HIV programs as well as Prevention of Mother To Child Transmission of HIV (PMTCT), HIV testing, and supporting long term retention in care (including defaulters tracing and providing appointment reminders). Ensuring quality performance of tasks by expert patients may increase their acceptability towards formal health workers. A recent review of the literature on task-shifting [23] demonstrates the need to note quality and safety concerns, professional and institutional resistance and also the need to sustain motivation and performance. Thus, governance, supervision, training and the role of remuneration for expert patients should also be considered as potential avenues for further research. Finally, cost-effectiveness studies should be conducted to explore the feasibility of scaling up expert patient programs broadly in high HIV prevalence, low-resource settings.

## Competing interest

The expert patient program at the time of study was funded by Paediatric AIDS Treatment for Africa.

## Authors’ contributions

Conceived and designed the study: LT, MvL; Implemented the intervention: AKC; Conducted the study: LT; Analyzed the data: LT; Wrote the paper: LT, MvL, FC, AKC, AM; Critically reviewed the manuscript; AKC, RB, AM. All authors approved the final manuscript.

## Pre-publication history

The pre-publication history for this paper can be accessed here:

http://www.biomedcentral.com/1472-6963/12/140/prepub
